# Gamma diversity and under-sampling together generate patterns in beta-diversity

**DOI:** 10.1038/s41598-021-99830-8

**Published:** 2021-11-02

**Authors:** Aniruddha Marathe, Dharma Rajan Priyadarsanan, Jagdish Krishnaswamy, Kartik Shanker

**Affiliations:** 1 Present Address: Ashoka Trust for Research in Ecology and the Environment (ATREE), Royal Enclave, Srirampura, Jakkur Post, Bangalore, 560064 India; 2 Centre for Ecological Sciences, Indian Institute of Science, Bangalore, 560012 India; 3 School of Environment and Sustainability, Indian Institute for Human Settlements, Bengaluru, India

**Keywords:** Ecology, Ecology

## Abstract

Beta diversity represents how species in the regional pool segregate among local communities and hence forms a link between local and regional species diversities. Therefore, the magnitude of beta diversity and its variation across geographic gradients can provide insights into mechanisms of community assembly. Along with limits on local or regional level diversities, effects of local abundance that lead to under-sampling of the regional species pool are important determinants of estimated beta diversity. We explore the effects of regional species pools, abundance distributions, and local abundance to show that patterns in beta diversity as well as the mean of species abundance distribution have distinct outcomes, depending on limits on species pools and under-sampling. We highlight the effect of under-sampling in some established relationships between gamma diversity and beta diversity using graphical methods. We then use empirical data on ant communities across an elevational gradient in the Eastern Himalayas to demonstrate a shift from effect of reduction in species pool to under-sampling at mid-elevations. Our results show that multiple processes with contrasting effects simultaneously affect patterns in beta diversity across geographic gradients.

## Introduction

Ecological communities have hierarchical structure; several species form local communities and a number of local communities form regional species pools^1^. At different scales of organization, multiple processes that may be deterministic or random limit species distribution, abundance and community composition^2^. Separating the effects of random and deterministic processes on community composition is an important objective in ecology. The presence of a species within a local community is limited by niche related deterministic processes such as climate and habitat suitability, along with biotic processes such as competition, neutral processes such as chance dispersal and anthropogenic impacts such as disturbance^3^. Outcomes of such processes summed across many species give rise to species diversity of any given local community or 'alpha diversity (α)'. The outcomes of species composition across local communities are not identical, hence diversity in the overall species pool or the 'gamma diversity (γ)' is generally much larger than alpha diversity. The variability in species composition between local communities that results in gamma diversity being larger than alpha diversity is defined as 'beta diversity (β)'^4^. Since beta diversity represents a link between local and regional diversities and represents how species in the regional pool are sorted among local communities, the magnitude of beta diversity and its variation across geographic gradients can provide inferences about community assembly along environmental gradients^5^.

Sharp gradients and variability in environmental conditions across a landscape can limit the distribution of species near its optimal niche and result in segregated distributions among habitats. Such deterministic variation in community composition is observed on mountains; for instance, most of the turnover among bird communities along elevational gradients in Costa Rica was due to habitat specialization^6^. Species found in relatively stable climates in the tropics are expected to evolve narrower niche breadths and therefore have narrow elevational range extents as well^7,8^. This results in finer subdivision of the landscape among species and therefore greater changes in species composition^6^. Such processes make alpha diversity increasingly smaller than gamma diversity and in turn, increases the beta diversity.

Measures of beta diversity can also change as a function of local abundance. Between two local communities that are part of the same regional species pool, the community with greater number of individuals will have a better representation of the regional pool. Hence, beta diversity will increase as local abundance decreases, all else being equal. These higher values are an artefact of under-sampling of the regional species pool^9^ rather than being the result of ecological processes that influence diversity directly. Many local processes such as disturbance, and fragmentation may limit local abundance and can have the same effect. For example, an increase in beta diversity with a decrease in fragment size has been observed for plants^10,11^, and small mammals^12^. A predominance of ‘drift related processes’ leading to chance colonization and extinction can also cause an increase in beta diversity^3^—a process that may be referred to as ecological under-sampling. Therefore, in order to understand the processes behind patterns in beta diversity, it is important to separate the effects of alpha diversity, gamma diversity, and under-sampling. In this paper, we extend interpretations from known relationships between gamma diversity and beta diversity in order to separate the effects of under-sampling, and further test for the effects of limits on alpha diversity in generating patterns in beta diversity using null models.

**Figure 1:**
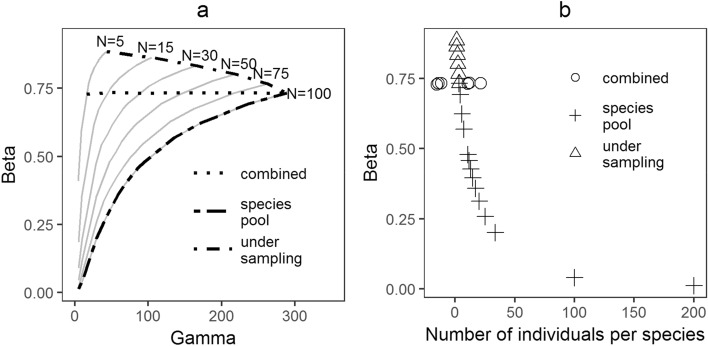
(**a**) The expected relationship between gamma diversity and beta diversity (redrawn based on Figure S2 and simulation algorithm by Kraft et al.^13^), which shows the relationship between beta diversity and gamma diversity obtained by sampling N individuals from a lognormal distribution. Each curve represents the effects of decreasing gamma diversity on beta diversity, while curves for smaller local abundances are steeper than for larger local abundances due to the effects of under-sampling of the regional pool. The three conditions highlighted in the figure are, 'under sampling' where there is a decrease in local abundance while gamma diversity is constant; 'combined' where gamma diversity and local abundance decrease simultaneously; and 'species pool' where there is a decrease in gamma diversity using the highest abundance values in the simulation. The dotted lines for 'under-sampling' and 'species pool' mark the two extremes of the simulation results and the area in between shows all possible values of beta diversity. 'Combined' is the special case where beta diversity is largely invariant across a large range of gamma diversity; (**b**) The relationship between the number of individuals per species and beta diversity obtained from the same simulation in (**a**). The open circles representing 'combined' have near identical values on the x-axis, but are drawn slightly separated only to ease the representation.

**Figure 2:**
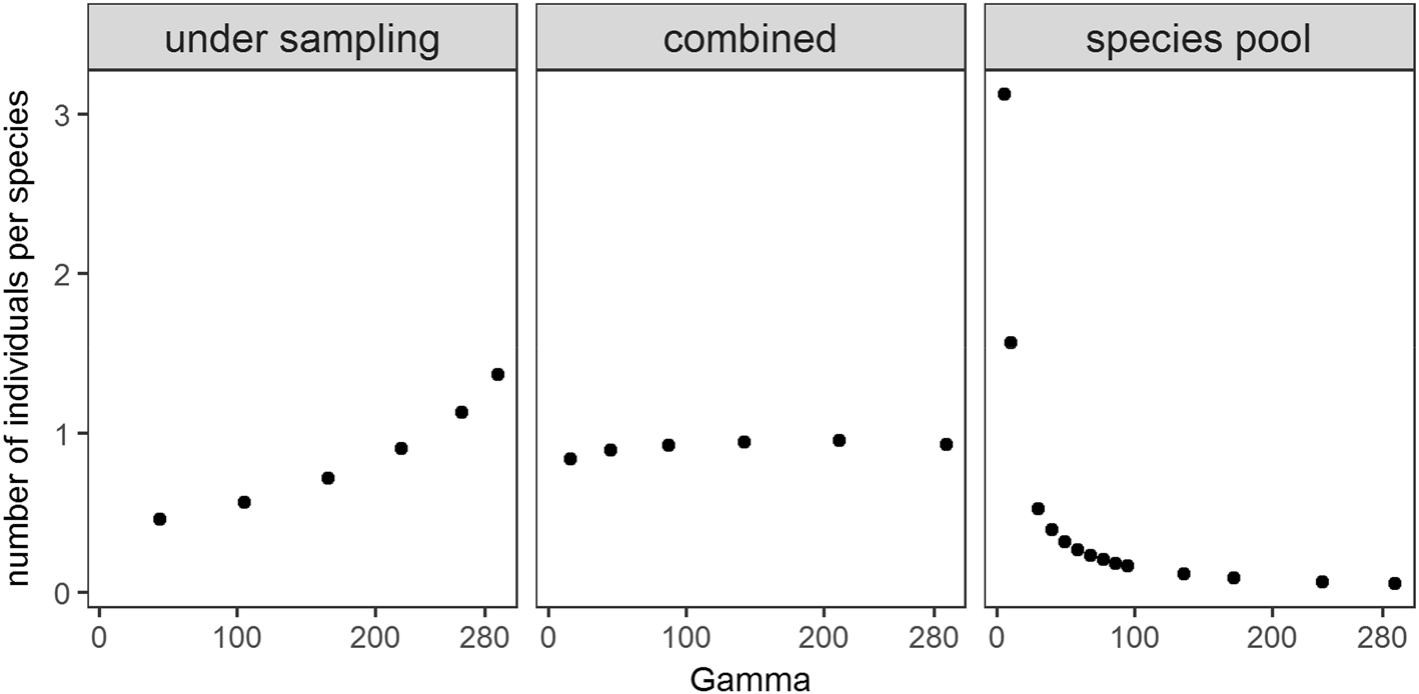
Variation in the number of individuals per species with observed species pool and under-sampling. The y-axis is scaled by subtracting each value from the mean.

To test the importance of limits on alpha diversity in generating geographic gradients in beta diversity, Kraft et al.^13^ simulated randomly assembled local communities from species pools of different sizes (10 to 280 species), so that local species richness was only limited by the sampling effect of the number of individuals in local communities (5 to 100 individuals). The simulation results show that: (1) beta diversity has an asymptotic relation with gamma diversity for a large range of local abundances (grey lines in Fig. 1a); (2) the estimate of beta diversity should increase when the same regional pool has smaller local abundances that are sampled, and (3) in a special case where the two processes counteract each other, beta diversity may remain unchanged over a large variation of observed gamma diversity depending on the relative rates of change in local abundances and the actual number of species in the regional species pool (Fig. 1a).

This shows that on real gradients, such as elevational or latitudinal gradients, beta diversity can change in different ways when there is a large variation in gamma diversity as well as local abundance. The expected beta diversity also shows a relationship with the number of individuals per species or the mean of the abundance distribution (Fig. 1b). This pattern has been used to suggest a stronger link between deterministic processes and beta diversity across latitudinal gradients^14^. However, since randomly sampled local communities also produce the same relationship, it is difficult to draw inferences about causal mechanisms from the negative correlation of beta diversity with the mean of the abundance distribution. In fact, the mean of the abundance distribution is an emergent property of the community that is the result of assembly processes, much like alpha or beta diversity. Variation in the mean of the abundance distribution with gamma diversity highlights this point. As in the case of beta diversity, the simulation results from Kraft et al.^13^ also show three distinct patterns for the relationship between the mean of the abundance distribution and gamma diversity, given the importance of under-sampling and species pools (Fig. 2). This shows that the relationship between beta diversity and the abundance distribution is an outcome of assembly processeses and patterns in either of the two variables can have diverse outcomes, under the combined influences of diversity in the regional species pool and under-sampling. Therefore, variation in the mean of the abundance distribution and beta diversity across gamma diversity (Fig. 2) reflects assembly processes better compared to just patterns in beta diversity across the mean of the abundance distribution (Fig. 1).

To empirically test these predictions, we studied ant communities on an elevational gradient in the Eaglenest Wildlife Sanctuary, India. The ant communities show a decrease in species diversity towards higher elevations^15^. The simulation results (Fig. 1) can apply to elevational gradients, where larger species pools represent low elevations and smaller species pools represent higher elevations. Therefore, the pattern in the two variables—the mean of the abundance distribution and beta diversity—across the elevational gradient should indicate the relative roles of species pool effects and under-sampling on beta diversity. In addition, limits on alpha diversity may vary across the elevational gradient, which will lead to a systematic variation in the difference between observed and null expectations of beta diversity. We test this using a null model to randomly assemble local communities from the species pool and apply it to empirical data on ant communities across the elevational gradient.

## Results

### Pattern of beta diversity across elevation

**Figure 3:**
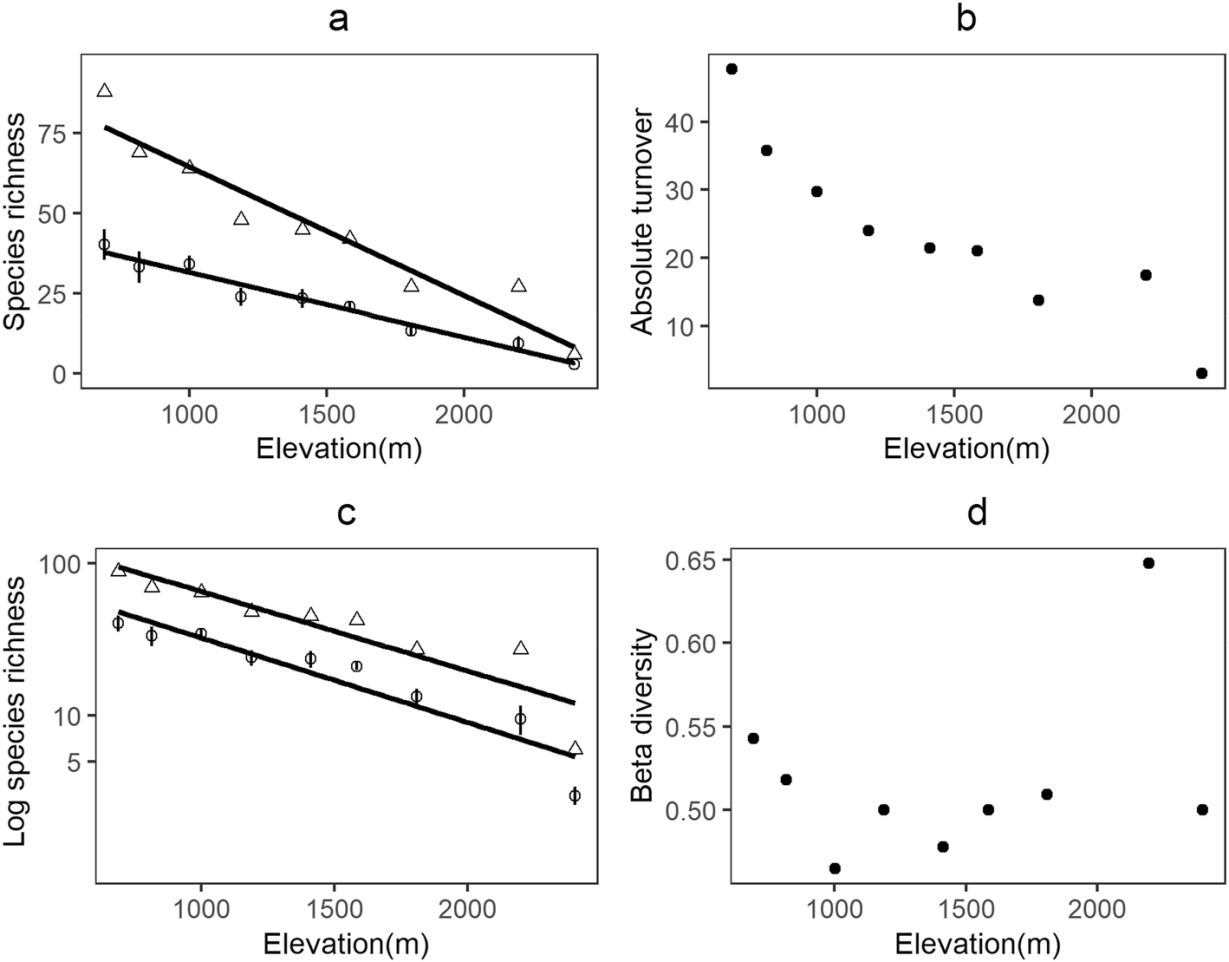
Variation in alpha and gamma diversity relative to each other with the corresponding measure of beta diversity. Triangles show gamma diversity while circles represent alpha diversity and vertical bars are standard error (**a**) alpha and gamma diversity across elevation; (**b**) absolute turnover across elevation; (**c**) log transformed alpha and gamma diversity across elevation; (**d**) multiplicative beta-diversity across elevation.

**Figure 4:**
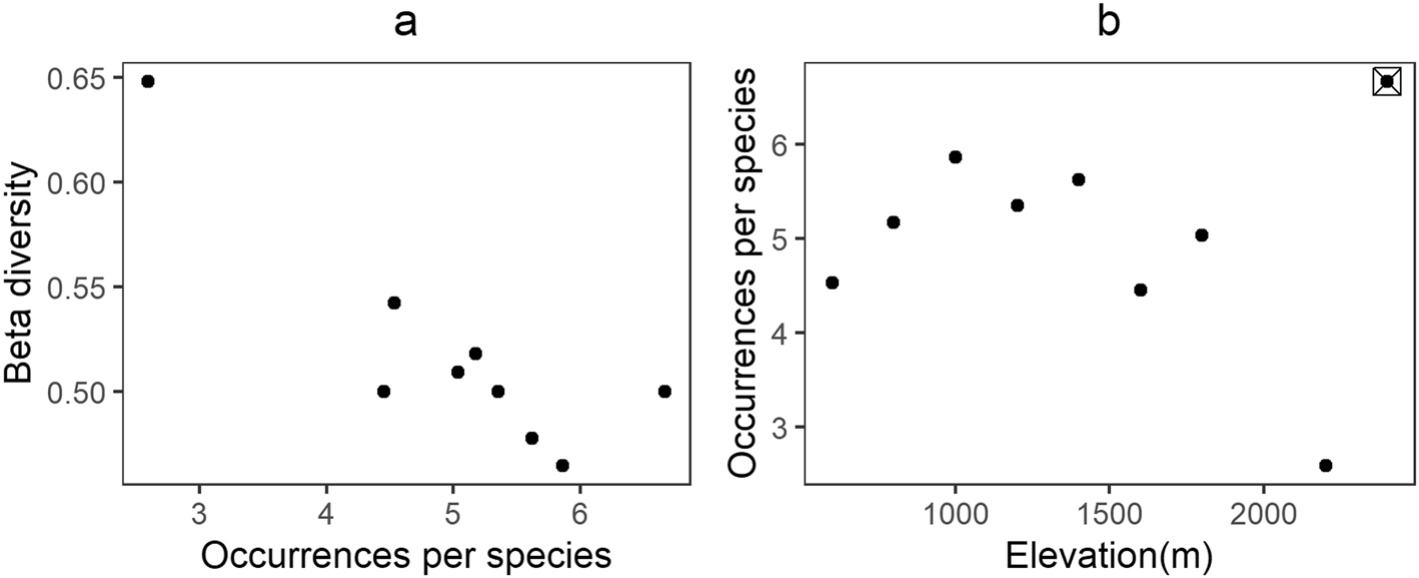
(**a**) Relationship between beta diversity and number of occurrences sampled per species; (**b**) Number of occurrences per species across elevation. The point marked with a cross inside a square box is considered as an outlier in this relationship (see discussion for further explanation).

The species richness of elevation bands or gamma diversity decreases across elevation with a steeper slope than the species richness of transects or alpha diversity (Fig. 3a). This represents the absolute turnover between transects within each elevation band (gamma–alpha or additive partitioning), which decreases with elevation (Fig. 3b). However, the two slopes are not different when the y-axis is log-transformed (Fig. 3c) and the corresponding multiplicative beta diversity is mostly invariant across elevations (Fig. 3d). Observed beta diversity decreases with an increase in the number of occurrences per species (Fig. 4a). The number of occurrences per species initially increases from lower elevations to 1400 m and then decreases towards higher elevation except for the highest elevation (ie.2400 m, Fig. 4b).

### Null expectations of beta diversity

**Figure 5:**
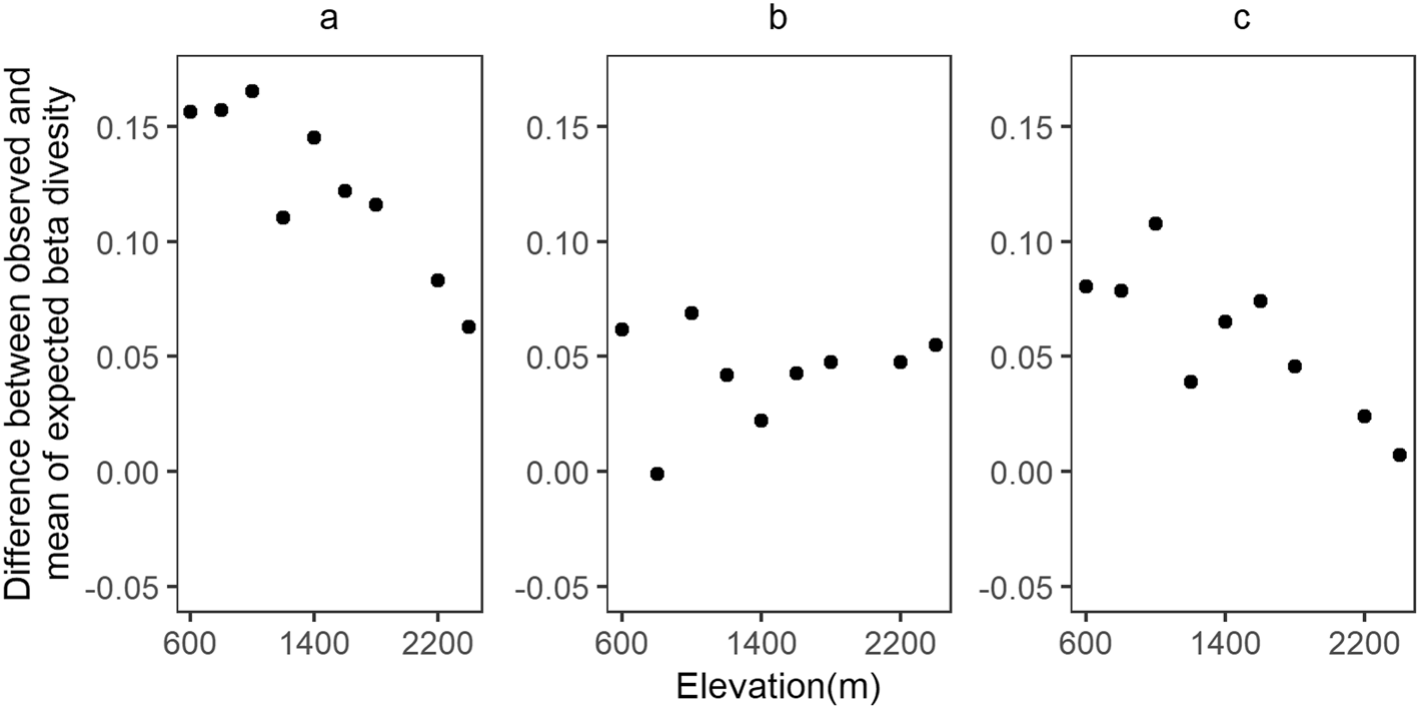
(**a**) Difference between observed and abundance based estimates of beta diversity; (**b**) Difference between observed and occurrence based estimates of beta diversity; (**c**) Difference between beta diversity estimates in panels ‘**a**’ and ‘**b**’.

The abundance as well as occurrence-based expectations of beta diversity obtained by random assembly of local communities were consistently smaller than observed values at all elevations, as shown by positive values of raw deviation (Fig. 5a, b). The aggregation corrected estimates of beta diversity, obtained by occurrence-based randomizations, were higher than the binomial or abundance-based model as expected and its deviation from the observed values was much smaller. Further, there was no pattern in the residuals of the aggregation expected beta diversity (Fig. 5b), while the difference between aggregation and binomial expectation decreased with elevation (Fig. 5c).

## Discussion

Beta diversity represents the link between local communities and the regional species pool. It is regarded as an important property of ecological communities that can help in providing inferences about mechanisms of community assembly. We revisit null models for beta diversity on environmental gradients and highlight multiple possibilities for null expectations, given the simultaneous effects of gamma diversity and ecological under-sampling. This analysis advances earlier works that focused on the deviation of observed values from the expected pattern. Here, we discuss expected patterns in beta diversity under different scenarios.

Redrawing the simulation results (Fig. 1) in Kraft et al.^13^ shows that the size of species pool and under-sampling can together lead to diverse null expectations. The results show that gamma diversity and under-sampling act in opposite ways and can potentially counter each other in terms of their effect on the pattern in beta diversity. Therefore, patterns in beta diversity need to be separated into cases where under-sampling dominates and where gamma diversity dominates or a combination of the two. This is possible as the three conditions produce distinct relationships between beta and gamma diversity.

There has been some debate about patterns in beta diversity across latitudinal gradients and the relevance of ecological processes in addition to under-sampling^9,14^. In the light of the findings of this study, a negative relationship between the mean number of individuals per species and gamma diversity^14^ should indicate a stronger effect of reduction in species pool on beta diversity, strengthening the original claim by Kraft et al.^13^.

The beta diversity pattern in the empirical data shows decreasing total turnover with elevation, but the pattern disappears in turnover proportional to gamma diversity (Fig. 3). A similar pattern is observed in some alpine communities as well^16,17^. This means that the rate of species turnover remains unchanged with variation in gamma diversity along the gradient. According to the null model, this points towards the effect of under-sampling counteracting the effect of decrease in gamma diversity. The unimodal pattern in the number of occurrences per species can be seen as a combination of two patterns with an initial increase up to mid elevations, and then a decrease towards higher elevations. Here, the highest elevation is considered an outlier, as a disproportionately small regional species pool (Fig. 3) could be potentially compensating the effects of under-sampling. As mentioned previously (Fig. 2), beta diversity at lower elevations could be limited by species pools or gamma diversity while at higher elevations, the effects of under-sampling become more important. Constraints on the number of gamma diversity estimates considered for this study (nine elevation bands) limits a more detailed statistical examination of the pattern, but it may be useful to examine similar patterns in future.

In the simulation results (Fig. 1), the number of species sampled in any local community and the resultant beta diversity are outcomes of a random distribution of individuals, and therefore should be analogous to expected values from the binomial model of species distribution^18^. Ecological limits on alpha diversity can cause the actual beta diversity to be higher or lower than the random expectation. Therefore, systematic changes in the difference between observed and expected values should indicate variation in the limits of alpha diversity along the gradient^5^. A number of studies explore this deviation along latitudinal, elevational, and other gradients^14,18–21^ where the observed beta diversity is consistently higher than its random expectation, which is also the case in this study. This indicates the effect of biologically relevant mechanisms limiting alpha diversity.

In the present study, the difference between the observed and random expectations of beta diversity decreased with elevation but within sample aggregation accounted most of this variation (represented by occurrence based estimates). While the difference between observed and aggregation accounted estimates are also positive, there is no systematic change with elevation (Fig. 5b). This shows that the importance of limits on alpha diversity is almost constant along the elevation gradient. This could be because transects in the study that represent local communities do not represent distinct local conditions or habitats, but are actually part of one continuous habitat and most of the differences occur with changes in elevation. A study where species compositional differences among distinct habitats are compared across elevational gradients may reveal changes in habitat filtering^22^. Lack of competitors due to independent environmental filtering and greater climatic variability at higher elevations could favour wider niches reducing the difference in species composition between habitat types towards higher elevations.

Considering the three results together, the decrease in gamma diversity, the pattern in the mean number of occurrences per species, and differences in the observed beta diversity and null expectations, we infer that beta diversity in ant communities on elevation gradients in Eaglenest Wildlife Sanctuary is driven by combination of a decrease in species pools, under-sampling, and the biological properties of species.

## Conclusion

Gradients in estimated beta diversity can be generated due to gradients in alpha diversity, gamma diversity, and its under-sampling through the influence of local abundance. As all three variables are likely to change simultaneously, identifying the exact drivers of beta diversity and linking it to community assembly is not straightforward. We show that beta diversity is expected to have distinct patterns under the influence of gamma diversity and local abundance. This also confirms earlier speculations about possible increases in beta diversity due to a reduction in local abundance under the influence of ‘ecological drift’.

Our results indicate that diverse patterns in the mean of the abundance distribution as well as beta diversity can appear by a combination of gamma diversity and random sampling and hence cannot be directly linked to additional community assembly mechanisms. Instead, the relationship between beta diversity and the mean of the abundance distribution across gamma diversity can be used to analyse processes of community assembly, as the patterns are distinct under conditions when either gamma diversity or under-sampling dominates. We then apply this framework to empirical data on ant communities from Eaglenest Wildlife Sanctuary to suggest a potential shift from limits on species pools to ecological under-sampling in driving beta diversity patterns along the elevational gradient.

## Materials and methods

### Study site and sampling methods

This study was conducted in the Eaglenest Wildlife Sanctuary (EWS) located in western Arunachal Pradesh, and is nested within the Himalayan mountain range, which is a region of global conservation importance^23^. EWS covers a wide elevation range from 500 to 3250 m. Ant communities were sampled within elevation bands of 200 m elevational width between 600 m and 2400 m, using methods similar to the Ants of Leaf Litter (ALL) protocol^24^. Each elevation band contained four sampling locations that represent local communities. Each local community was sampled using ten trapping stations which consisted of Winker extractors and pitfall traps arranged on a 100 m transect at 10 m intervals (see^15^ for details of study site, and sampling methods).

### Analysis of beta diversity

We define alpha diversity as mean species richness of all four local samples, and gamma diversity as the total observed species richness of each elevation band. We represent beta diversity as the multiplicative partition (beta = 1 − mean alpha/gamma) within each elevation band. To visualize the pattern in beta diversity across elevation, we used scatter plots for alpha and gamma diversity across elevation. The difference between slopes for alpha and gamma on a linear scale represent absolute turnover. When the y-axis is log transformed, the difference represents proportional turnover^23^.

To examine the relative effects of decrease in gamma diversity and abundance on beta diversity, we used the pattern in the number of occurrences (number of traps from which a species is recorded) per species across elevations. Here, we use occurrence instead of abundance as it is a better measure of commonness and rarity for ants^25^. The distribution of foraging worker ants is very likely aggregated, as ants are social insects that forage in trails or groups of workers. In addition, the proximity of traps to ant nests may cause a greater number of captures in certain traps^26^. Either can lead to an aggregated distribution of individuals among traps, which would reduce the number of traps occupied by each species compared to the random distribution. However, it is not straightforward to separate the biological and sampling related causes of aggregation. Therefore, we used occurrence data for all analyses.

In order to test if there are systematic changes in the limits on alpha diversity that lead to beta diversity across elevational gradients, we used null models that randomly assembled local communities to generate expected values of beta diversity. In earlier uses of such null models, each local community was assembled by sampling the observed number of individuals without replacement from the abundance distribution of the species pool. Hence, the relative commonness and rarity of species and the abundance of local communities are the same as the observed data, but species are distributed in local communities randomly^13,18^. Such models represent the expected beta diversity under the binomial model of species distributions^18^. The magnitude of difference between observed and random expectation of beta diversity indicates the strength of mechanisms in addition to random sampling. Systematic variation in this difference indicates a gradient in the importance of random vs. deterministic processes on beta diversity. We assembled null communities in the same manner as Kraft et al.^13^, but used occurrence data in addition to abundance, for the reasons explained earlier. During randomization, the total number of times a species is recorded at an elevation band is the same as observed values but its distribution among local communities is randomized, with an additional constraint that the maximum occurrence of species does not exceed the number of traps available. We performed 1000 iterations of the null model; for each iteration, we calculated alpha diversity and the resulting beta diversity. Mean values of all iterations were used as expected beta diversity from the null model.

We used Standardized Effect Sizes (SES) as well as the raw difference between mean and observed values as a measure of deviation as the two can have contrasting patterns^14^. All analyses were carried out in R v4.0.2 statistical software^27^.

## Supplementary Information

Supplementary Information 1.

Supplementary Information 2.

## Data Availability

The data used in this study is available as supplementary material.
