# Supplementary material for: Gamma diversity and under-sampling together generate patterns in beta-diversity
Source: Sci Rep. 2021 Nov 2;11:21420. doi: 10.1038/s41598-021-99830-8 (PMC8563776; doi:10.1038/s41598-021-99830-8)
Supplement: Supplementary file 1 — Supplementary Information 1. [file 41598_2021_99830_MOESM1_ESM.docx]

**Gamma diversity and under-sampling together generate patterns in beta-diversity**

**Aniruddha Marathe^1,2*^_,_ Dharma Rajan Priyadarsanan^1^, Jagdish Krishnaswamy^1^ and Kartik Shanker^1,2^**

^1^ Ashoka Trust for Research in Ecology and the Environment (ATREE), Royal Enclave, Srirampura, Jakkur post, Bangalore - 560064, India.

^2^ Centre for Ecological Sciences, Indian Institute of Science, Bangalore - 560012, India.

^*^Corresponding author: [aniruddha.pravin.marathe@gmail.com](mailto:aniruddha.pravin.marathe@gmail.com)

**Supplementary Table 1**

| Elevation | Mean species richness (alpha diversity) | SD of species richness | Pooled species richness (gamma diversity) | Beta diversity | Number of occurrences recorded |
| --- | --- | --- | --- | --- | --- |
| 600 | 40.25 | 9.53 | 88 | 0.54 | 399 |
| 800 | 33.25 | 9.67 | 69 | 0.51 | 357 |
| 1000 | 34.25 | 5.12 | 64 | 0.46 | 375 |
| 1200 | 24 | 5.59 | 48 | 0.50 | 257 |
| 1400 | 23.50 | 5.91 | 45 | 0.47 | 253 |
| 1600 | 21 | 2.44 | 42 | 0.50 | 187 |
| 1800 | 13.25 | 2.30 | 27 | 0.50 | 136 |
| 2200 | 9.50 | 4.12 | 27 | 0.64 | 70 |
| 2400 | 3 | 0.81 | 6 | 0.50 | 40 |

**Details of supplementary files**

**1. SupplementaryMaterial-2:** The file contains species distribution information across elevations and replicates. Details of the headers are as follows:

| Header | Detail |
| --- | --- |
| Area | Abbreviation for larger area of sampling. Data presented here are from a single area |
| elev_Zone | code for elevation zones. Values from 3 to 12 |
| replicate | code for replicates within elevation zones. Unique values in the column: A,B,C,D |
| method | Method of trapping. P for pitfall trap, and W for Winkler |
| season | Season- code M for monsoon. Data presented here were collected within a single season |
| year | Year of sampling. All the Data presented here are from 2013 only |
| trap_No | identifier of the trap at each replicate |
| transect | combination of Area, elev_zone, replicate, method, season, year |
| sample_ID | combination of Area, elev_zone, replicate, method, season, year, trap id |
| elevation | Elevation in meters of each elevation zone |
